# Neuroprotective effects of phenolic antioxidant tBHQ associate with inhibition of FoxO3a nuclear translocation and activity

**DOI:** 10.1111/j.1471-4159.2012.07877.x

**Published:** 2012-10

**Authors:** Parmvir K Bahia, Victoria Pugh, Kimberley Hoyland, Victoria Hensley, Marcus Rattray, Robert J Williams

**Affiliations:** * Wolfson Centre for Age-Related Diseases, King’s College LondonLondon, UK; † Reading School of Pharmacy, University of ReadingUK; ‡ Department of Biology and Biochemistry, University of BathUK

**Keywords:** caspase, dietary antioxidant, Fas ligand, motor neuron, NMDA receptor, PI3-kinase

## Abstract

The Forkhead transcription factor, FoxO3a induces genomic death responses in neurones following translocation from the cytosol to the nucleus. Nuclear translocation of FoxO3a is triggered by trophic factor withdrawal, oxidative stress and the stimulation of extrasynaptic NMDA receptors. Receptor activation of phosphatidylinositol 3-kinase (PI3K)–Akt signalling pathways retains FoxO3a in the cytoplasm, thereby inhibiting the transcriptional activation of death-promoting genes. We hypothesized that phenolic antioxidants such as tert-Butylhydroquinone (tBHQ), which is known to stimulate PI3K–Akt signalling, would inhibit FoxO3a translocation and activity. Treatment of cultured cortical neurones with NMDA increased the nuclear localization of FoxO3a, reduced the phosphorylation of FoxO3a, increased caspase activity and up-regulated Fas ligand expression. In contrast the phenolic antioxidant, tBHQ, caused retention of FoxO3a in the cytosol coincident with enhanced PI3K- dependent phosphorylation of FoxO3a. tBHQ-induced nuclear exclusion of FoxO3a was associated with reduced FoxO-mediated transcriptional activity. Exposure of neurones to tBHQ inhibited NMDA-induced nuclear translocation of FoxO3a, prevented NMDA-induced up-regulation of FoxO-mediated transcriptional activity, blocked caspase activation and protected neurones from NMDA-induced excitotoxic death. Collectively, these data suggest that phenolic antioxidants such as tBHQ oppose stress-induced activation of FoxO3a and therefore have potential neuroprotective utility in neurodegeneration.

The mammalian homologue, class O of forkhead box transcription factors (FoxO) comprises four members (Fukunaga *et al.* 2005): *FoxO1*, *FoxO3*, *FoxO4* and *FoxO6,* and of these *FoxO1*, *FoxO3* and *FoxO6* expression has been detected in the brain (Furuyama *et al.* 2000; Biggs *et al.* 2001; Hoekman *et al.* 2006). FoxO binds to a core DNA consensus sequence, referred to as the Forkhead-response element (FHRE) to increase expression of pro-apoptotic genes (Furuyama *et al.* 2000). While FoxO activity is essential to normal programmed cell death during development, it has also been implicated in initiating apoptosis in cases of brain injury and in the progression of neurodegenerative disorders in the mature nervous system (Chong *et al.* 2005; Fukunaga *et al.* 2005; Su *et al.* 2009). There are several stressful triggers that stimulate nuclear translocation of FoxO in neurones including trophic factor withdrawal (Gilley *et al.* 2003; Barthelemy *et al.* 2004; Biswas *et al.* 2007), oxidative stress (Lehtinen *et al.* 2006), ischaemia (Fukunaga *et al.* 2005) and the activation of extrasynaptic NMDA receptors (Dick and Bading 2010). FoxO subsequently increases expression of genes encoding death proteins such as Bim (Bcl-2-interacting mediator of cell death) and Fas ligand (Brunet *et al.* 1999; Gilley *et al.* 2003; Barthelemy *et al.* 2004; Biswas *et al.* 2007).

Transcriptional activity of FoxO is partly determined by its subcellular location, which in turn is regulated by phosphorylation of three key residues at Thr-24, Ser-256 and Ser-319 (Nakae *et al.* 1999) lying downstream of PI3K–Akt signalling. FoxO3a phosphorylation at these sites causes it to be exported from the nucleus and retained in the cytoplasm in a Crm-1- and 14-3-3-dependent manner, thus rendering FoxO3a incapable of transcribing target apoptotic genes (Brunet *et al.* 1999). Neuroprotective phenolic antioxidants such as tBHQ and dietary flavonoids stimulate PI3K–Akt signalling and up-regulate Nrf2-dependent antioxidant response element activity (Johnson *et al.* 2002; Kraft *et al.* 2004; Schroeter *et al.* 2007; Bahia *et al.* 2008; Arredondo *et al.* 2010; Williams and Spencer 2012), but it is not known if they also inhibit FoxO3a activity to exert neuroprotective effects in neurones. The aim of this study, therefore, was to establish a link between FoxO3a activity and NMDA receptor-evoked neuronal cell death and to determine if phenolic antioxidants such as tBHQ could exert neuroprotection by opposing activation of this pro-apoptotic pathway. The work described here demonstrates that exposure of neurones to tBHQ inhibited NMDA-induced nuclear translocation of FoxO3a, prevented NMDA-induced up-regulation of FoxO-mediated transcriptional activity, blocked caspase activation and protected neurones from NMDA-induced excitotoxic death.

## Materials and methods

### Materials

NMDA and kainate were purchased from Tocris Bioscience (Bristol, UK). Tert-butyl hydroquinone (tBHQ) was purchased from Sigma-Aldrich (Poole, UK) and wortmannin from Calbiochem Corporation (La Jolla, CA, USA). Primary antibodies used were anti-FoxO3a and anti-phospho FoxO3a (Cell Signaling Technology, Danvers, MA, USA), anti-Fas ligand from Millipore (Watford, UK), anti-activated caspase 3 (Asp175) from New England Biolabs (Hitchin, UK) anti-beta Tubulin from Abcam (Cambridge, UK), anti-SMI-32R from Covance (Princeton, NJ, USA). Fluorescently labelled secondary antibodies, chicken anti-rabbit Alexa Fluor® 488 and goat anti-mouse Alexa Fluor® 568 were obtained from Invitrogen (Paisley, UK) and horseradish peroxidase-conjugated goat anti-rabbit antibody from Millipore. Cells were mounted in Mowiol® mounting medium with Hoechst 33342 (Calbiochem Corporation). The Dual-Glo luciferase assay system, pGL3 basic, pRL-TK renilla and restriction enzymes were from Promega (Madison, WI, USA). Cell culture reagents were obtained from Invitrogen and all other reagents from Sigma-Aldrich or Merck.

### Cell culture

Animals were maintained and used according to the U.K. Animals (Scientific Procedures) Act, 1986 and institutional ethical approval. Animals were killed using cervical dislocation (Schedule 1 procedure) according to Home Office guidelines. Primary cultured neurones were prepared from 15 to 16-day-old Swiss mouse embryos (NIH, Harlan, UK).

#### Cortical neurone culture

Cortical neurones were prepared essentially as described previously (Hoey *et al.* 2009). Briefly, dissociated cortical neurones were plated at 10^6^ cells/mL into 24-well or six-well Nunc multiwell plates (Nunc, Naperville, IL, USA) that had been coated previously overnight with 15 μg/mL poly l-ornithine (Sigma-Aldrich). Neurones were seeded in phenol red-free Neurobasal B27-supplemented medium (Invitrogen). Cells were cultured at 37°C in a humidified atmosphere of 95% air and 5% CO_2_ and experiments were performed at 7–9 DIV. Neurones for immunocytochemistry were cultured on 13-mm coverslips pre-coated with poly l-ornithine and 2 μg/mL laminin (Sigma-Aldrich).

#### Motor neurone culture

Motor neurones were prepared essentially as described previously (Rainey-Smith *et al.* 2010). Spinal cords from embryos were dissected out into phosphate-buffered saline containing 0.6% glucose (PBS-gluc). The meninges were removed and the cords cut into ∼5 mm^3^ pieces. Up to five cords in 1.5 mL PBS-gluc were transferred to 15 mL centrifuge tubes. Trypsin (Invitrogen) was added to a form a final concentration of 0.05%. The tubes were then incubated at 37°C for 10 min with gentle agitation every 2 min. The supernatant was removed and the tissue resuspended in 1 mL L-15 medium containing 0.4% w/v bovine serum albumin (BSA) and 0.1 mg/mL DNase (D2; Worthington Biochemical Corporation, Lakewood, NJ, USA). Tissue was then agitated for 3 min, triturated and the supernatant transferred to a clean centrifuge tube. The pellet was then resuspended in 1 mL L-15 containing 0.4% w/v BSA and 20 μg/mL Dnase and triturated again and the supernatant collected. This process was repeated and the pooled supernatants were then transferred onto a BSA cushion (1.5 mL of L-15 containing 4% w/v BSA for the equivalent of five spinal cords) and spun for 5 min at 500 *g* in a Heraeus Biofuge Primo centrifuge (DJB Labcare, Bucks, UK). The supernatant was then removed and the pellet resuspended in 1 mL of culture medium consisting of phenol red-free Neurobasal™ Medium supplemented with glutamine (2 mM), streptomycin (100 μg/mL and penicillin 100 u/mL) and B27 diluted 1 : 50. This was carefully layered onto 1.5 mL OptiPrep® density gradient medium (Axis-Shield UK, Cambridge, UK), diluted in L-15 to contain 10.4% w/v iodixanol, in a 15 mL centrifuge tube. The cells were then spun at 1000 *g* for 15 min with the centrifuge brake switched off. Cells visible at the medium/OptiPrep® interface were collected and spun through a second BSA cushion for 5 min at 1500 rpm. Between 20 000 and 30 000 cells were resuspended per millilitre of culture medium and strained through a 40 μm nylon strainer (VWR International Ltd, Leicestershire, UK). Cells were then plated out onto 13 mm coverslips coated with poly l-ornithine (1 h at 20°C) followed by a second incubation with culture medium containing 2 μg/mL laminin (Sigma-Aldrich).

### Plasmid constructs

*pFHRE-luc:* Synthesized oligos containing three repeats of the insulin response element (as described by Guo *et al.* 1999) were obtained from Sigma-Aldrich. The element is shown underlined and referred to as the Forkhead-Response Element or FHRE; forward: 5′-CAAGCAAAACAAGCTAGCAAAACAAGCTAGCAAAACAAGTA-3′, reverse: 5′- AGCTTACTTGTTTTGCTAGCTTGTTTTGCTAGCTTGTTTTGCTTGGTAC-3′). These were 5′-phosphorylated and annealed in a buffer consisting of 10 mM Tris–HCl, 50 mM NaCl and 1 mM EDTA for 5 min at 95°C and then cooled to 20°C. Annealed oligos were ligated into the SacI and HindIII sites of the pGL3 basic vector (Promega). *pARE-luc:* The pARE vector construction was described in detail previously (Bahia *et al.* 2008). Briefly, synthesized oligos were annealed as described above and ligated into the pGL3 basic vector via the SacI and HindIII sites.

### Transfection

Neurones in 24-well plates (5 × 10^5^ cells/well) were transfected using either pARE-luc or pFHRE-luc cis reporter plasmids along with pRL-TK renilla. Transfections were performed using 0.5 μg DNA/well and 0.5 μL LipofectAMINE 2000 (Invitrogen) according to the manufacturer’s instructions).

### Luciferase assay

Cells cultured in 24-well plates were treated by adding compounds directly to the media, at the different time points indicated, before the assay. The assay was performed according to the manufacturer’s instructions (Promega). Briefly, medium was aspirated and cells left in 20 μL Glo Lysis buffer for 10 min. Lysates were transferred to a 96-well luminometer plate (Sigma-Aldrich). Luciferase activities produced by the luciferase reporter plasmids were measured by adding 20 μL of luciferase substrate, then using a Veritas microplate luminometer (Turner Biosystems Inc., Sunnyvale, CA, USA). All treatments were performed in triplicate on three to four independent cultures. Firefly luciferase activities were standardized to the corresponding Renilla luciferase activities and statistical analyses performed using one-way anova and the Bonferroni *post hoc* test.

### Immunoblotting

Cortical neurones were cultured in six-well Nunc™ plates, the medium aspirated and the cells then lysed in 100 μL sodium dodecyl sulphate–polyacrylamide gel electrophoresis sample buffer (62.5 mM Tris, pH 6.8, 2% SDS, 5% 2-mercaptoethanol, 10% glycerol and 0.0025% bromophenol blue) containing Complete, Mini; Protease Inhibitor Cocktail Tablets from Roche Applied Science (Burgess Hill, UK). Samples were then boiled for 5 min. Thirty microlitres of the samples were resolved by 9% Tris-glycine sodium dodecyl sulphate–polyacrylamide gel electrophoresis with running buffer consisting of 25 mM TRIS-base, 192 mM glycine and 0.1%SDS. After gel electrophoresis, proteins were transferred to Hybond nitrocellulose (GE Healthcare, Piscataway, NJ, USA). Immunodetection of phospho-FoxO3a and FoxO3a was performed using primary polyclonal antibodies diluted at 1 : 200 and Fas ligand at 1 : 1000. β-Tubulin levels were assessed as loading controls at an antibody dilution of 1 : 10 000. The horseradish peroxidase-conjugated goat-anti-rabbit IgG secondary antibody was used at 1 : 10 000. Membranes were blocked for 30 min in TRIS-buffered saline containing 4% milk powder and then washed 2 × 5 min in TBS containing 0.05% Tween-20. Primary antibodies were made up in 1% milk powder in TBS containing 0.05% Tween-20 and incubated overnight. Bands for the phospho-FoxO3a (ser318/321) antibody were detected using the ECL plus system, and all other antibodies using the ECL reagent (GE Healthcare) followed by exposure to Hyperfilm ECL according to the manufacturer’s instructions (GE Healthcare) for 1–10 min to ensure signal linearity and developed. Bands were analysed using Image-J quantifier software (Wayne Rasband, NIH, MD, USA) and band optical density expressed as a ratio relative to protein loading control. Data were analysed using GraphPad Prism software (GraphPad Software San Diego, CA, USA), and treatment group compared to control using an unpaired *t*-test.

### Immunocytochemistry

Treatments were performed by adding compounds directly to the medium as described prior to fixing. Neurones were washed in PBS (NaCl 136.9 mM, KCl 2.7 mM, Na_2_HPO_4_ 9.2 mM, KH_2_PO_4_ 1.8 mM, pH 7.2), then fixed in PBS containing 4% paraformaldehyde (Sigma-Aldrich) for 10 min. Cells were left in antibody blocking solution [PBS containing 2% bovine serum albumin and 2% horse serum (Sigma-Aldrich) and 0.2% Triton X-100 (Merck)] for 1 h. They were then incubated in antibody blocking solution containing 1 : 200 dilution of anti-FoxO3a antibody (Santa Cruz Biotechnologies, Santa Cruz, CA, USA) for 4 h. Cells were given three 5-min washes in PBS containing 0.1% Tween 20 (Merck). A second incubation using a chicken anti-rabbit Alexa Fluor 488 antibody (Invitrogen) was performed for 1 h. Cells were washed again in PBS containing Tween-20, then incubated for 5 min in PBS containing 10 μM Hoescht (2′-(4-Ethoxyphenyl)-5-(4-methyl-1-piperazinyl)-2,5′-bi-1H-benzimidazole, 3HCl HOE 33342 from Merck Chemicals Ltd, Hull, UK) and the coverslips mounted on glass slides using Mowiol 4-88 hard-setting mounting medium (Sigma). Staining results were viewed on a Zeiss apotome microscope and recorded using Axiovision LE software (Carl Zeiss Ltd., Hertfordshire, UK). For quantitative immunocytochemistry, cells were immunostained for FoxO3a and active caspase 3 following exposure to NMDA (50 μM) and/or tBHQ (30 μM) for 2–18 h. Three separate experiments were performed with six to nine independent fields analysed for each condition at each time point. Images were captured using fluorescence microscopy (Zeiss Axioimager A1) and nuclei (Hoe 33342 positive) were manually counted for each field. There were 50.0 ± 2.1 nuclei per field (*n* = 138) and no significant differences between the nuclear number with treatment or time. For activated caspase 3, the number of nuclei surrounded by positive (+) or very strong (++) cytoplasmic staining were counted and these two populations were combined for analysis. For foxO3A, the number of nuclei with positive staining in the nucleus were counted. Data were analysed using GraphPad Prism software (GraphPad Software San Diego) at each time point, using one-way anova followed Bonferonni’s Multiple Comparison test.

## Results

### NMDA causes pronounced dephosphorylation and nuclear accumulation of FoxO3a

**Fig 1:**
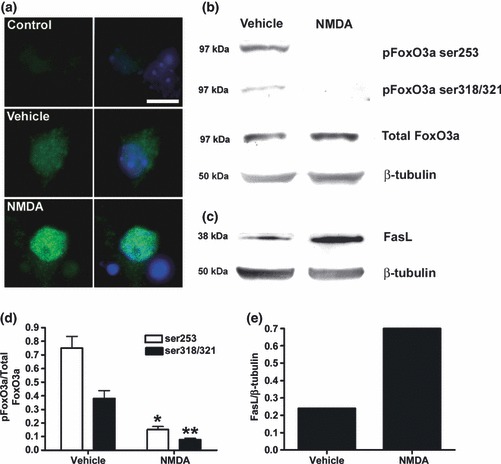
NMDA inhibits FoxO3a phosphorylation, stimulates FoxO3a nuclear translocation and up-regulates FasL. Cortical neurones were treated with NMDA (50 μM) for 2 h, and FoxO3a phosphorylation and FoxO3a translocation measured by immunoblotting (b, d) and immunocytochemistry, respectively, (a) and FasL expression by immunoblotting only (c, e). Reduced dephosphorylation of both pFoxO3a ser^253^ and pFoxO3a ser^319/321^ was evident following exposure to NMDA (b, d) with no change in total levels of FoxO3a or β-tubulin, but was associated with an increase in the levels of FasL (c, e). Blots shown are representative of three independent experiments. (d) Data presented as mean band intensity (mean ± SEM, *n* = 3) expressed as pFoxO3a relative to total FoxO3a following exposure to Vehicle control or NMDA (*p < 0.05, **p < 0.01). (e) Data presented as mean band intensity (mean ± SEM, *n* = 3) expressed as FasL relative to β-tubulin following exposure to Vehicle control or NMDA. To determine whether NMDA-mediated dephosphorylation of FoxO3a resulted in the nuclear accumulation of FoxO3a, cells were probed with an anti-FoxO3a antibody [(a), left and right panels] and counter-stained with Hoe 33342 (right panel only) to label nuclei. Control represents staining because of secondary antibody only. Under basal conditions, FoxO3a was distributed throughout the cytoplasm of cortical neurones with some diffuse staining evident in the nucleus (a, Vehicle). Following treatment with NMDA, FoxO3a staining was almost exclusively nuclear (a, NMDA). Scale bar = 20 μm.

FoxO3a is regulated by phosphorylation at threonine^32^ (T1) and serine^253/315^ residues (S1 and S2 respectively). Akt-mediated phosphorylation of S1 is necessary for subsequent phosphorylation at the other sites (Nakae *et al.* 2000) and the absence of S1 phosphorylation results in unmasking of the FoxO3a nuclear localization signal (Fukunaga *et al.* 2005). We first determined whether excitotoxic stimuli reduce FoxO3a phosphorylation and drive nuclear accumulation of FoxO3a in neurones. Cortical neurones were treated with NMDA (50 μM) for 2 h, and FoxO3a phosphorylation and FoxO3a translocation measured by immunoblotting and immunocytochemistry. There was significant dephosphorylation of both the S1 (pFoxO3a ser^253^) and S2 (pFoxO3a ser^319/321^) sites by 80% and 73%, respectively, relative to total FoxO3a levels following exposure to NMDA (Fig 1b and d) with no change in total levels of FoxO3a or β-tubulin (Fig 1b). To determine whether NMDA-mediated dephosphorylation of FoxO3a resulted in the nuclear accumulation of FoxO3a cells were probed with an anti-FoxO3a antibody (Fig 1a). Under control conditions FoxO3a was distributed throughout the cytoplasm of cortical neurones with some diffuse staining evident in the nucleus (Fig 1a, Vehicle). Following treatment with NMDA, FoxO3a staining was almost exclusively nuclear (Fig 1a, NMDA). Kainate induced a similar redistribution of FoxO3 from cytoplasm to nucleus in cortical neurones (not shown) and also in motor neurones (Figure S1).

We next examined Fas ligand (FasL) expression. FasL is a member of the tumour necrosis factor (TNF) family, which binds to the Fas receptor to initiate cell death via caspase signalling cascades (e.g. Raoul *et al.* 1999). The FasL gene has three FHRE (Brunet *et al.* 1999), and induction of FasL by FoxO3A has been linked directly with neuronal death (Barthelemy *et al.* 2004). To determine whether excitotoxic signalling to FoxO3a resulted in increased expression of FasL, immunoblotting was performed on lysates from cortical neurones treated with NMDA. Low levels of FasL protein could be detected under control conditions and exposure to NMDA (50 μM, 2 h) caused a two-fold increase in FasL (Fig 1c and e), suggesting that following translocation to the nucleus, FoxO3a up-regulates FasL in cortical neurones.

### tBHQ enhances phosphorylation and cytoplasmic retention of FoxO3a

**Fig 2:**
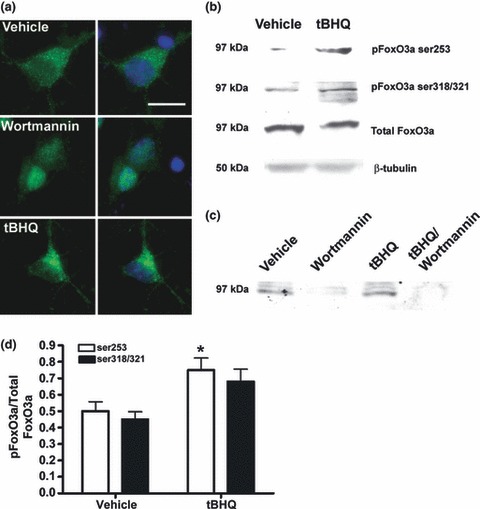
tert-Butylhydroquinone (tBHQ) stimulates FoxO3a phosphorylation and promotes cytosolic accumulation of FoxO3a. Cortical neurones were treated with tBHQ (30 μM; 2 h), or wortmannin (150 nM; 2 h) and FoxO3a phosphorylation and FoxO3a translocation were measured by immunoblotting and immunocytochemistry. tBHQ increased the phosphorylation of both the S1 (pFoxO3a ser^253^) and S2 (pFoxO3a ser^319/321^) sites without changing the total levels of FoxO3a or β-tubulin (b, d). Blots shown are representative of three independent experiments. (d) Data presented as mean band intensity (mean ± SEM, *n* = 3) expressed as pFoxO3a relative to total FoxO3a following exposure to Vehicle control or tBHQ (*p < 0.05). Basal (vehicle) and tBHQ-induced phosphorylation of pFoxO3a ser^253^ was abolished by wortmannin (c). To determine whether tBHQ-induced phosphorylation of FoxO3a affected the cellular distribution of FoxO3a cells were probed with an anti-FoxO3a antibody [(a) left and right panels] and counter-stained with Hoechst 33342 (right panel only) to label nuclei. Under control conditions, FoxO3a was distributed throughout the cytoplasm of cortical neurones with some low-level diffuse staining evident in the nucleus (Fig 2, Vehicle). Following treatment with tBHQ, FoxO3a staining was strongly cytoplasmic (Fig 2, tBHQ). This was particularly evident when compared with cells treated with the PI3K inhibitor wortmannin, which resulted in pronounced nuclear accumulation of FoxO3a (Fig 2, Wortmannin). Images shown are representative of 100 individual cells. tBHQ caused strong cytoplasmic accumulation of FoxO3a in 45% of stained cells. Scale bar = 20 μm.

Having demonstrated that NMDA caused FoxO3a dephosphorylation and translocation to the nucleus, and induced FasL, we wished to determine whether neuroprotective phenolic antioxidants such as tBHQ could suppress FoxO3a activation. It was not possible to demonstrate tBHQ inhibition of FasL expression as the basal levels in neurones were too low (see Fig 1c). Cortical neurones were treated with tBHQ (30 μM, 2 h), and FoxO3a phosphorylation and FoxO3a translocation were measured by immunoblotting and immunocytochemistry. tBHQ induced a clear trend towards increased phosphorylation of both the S1 (pFoxO3a ser^253^) and S2 (pFoxO3a ser^319/321^) sites by 149% and 151%, respectively, relative to total foxO3a levels without changing the total levels of FoxO3a or β-tubulin (Fig 2b). To determine whether tBHQ-mediated phosphorylation of FoxO3a affected the cellular distribution of FoxO3a, cells were probed with an anti-FoxO3a antibody (Fig 2a). Under control conditions, FoxO3a was distributed throughout the cytoplasm of cortical neurones with some low-level diffuse staining evident in the nucleus (Fig 2, Vehicle). Following treatment with tBHQ, FoxO3a staining was strongly cytoplasmic (Fig 2, tBHQ). This was particularly evident when compared with cells treated with the PI3K inhibitor, wortmannin, which resulted in pronounced nuclear accumulation of FoxO3a (Fig 2, Wortmannin). Wortmannin abolished basal (vehicle) and tBHQ-mediated FoxO3a phosphorylation (Fig 2c) and prevented tBHQ-induced cytoplasmic retention of FoxO3a (not shown).

### tBHQ enhances antioxidant response element- and inhibits forkhead-response element- activity in neurones

**Fig 3:**
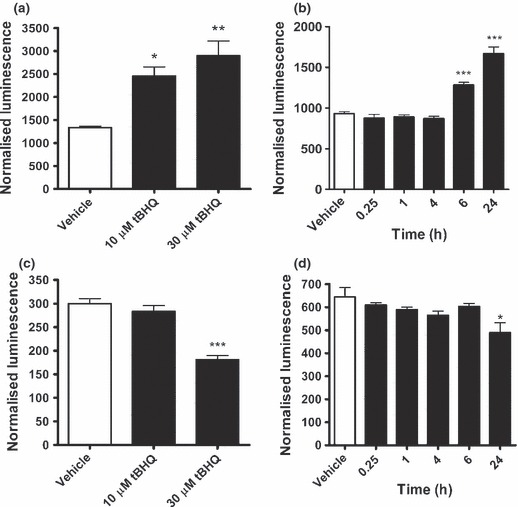
Inhibition of FKHD-mediated gene expression by tert-Butylhydroquinone (tBHQ). Cortical neurones (8 DIV) were transfected with pARE-luc (a, b) or FHRE-luc (c, d) and then treated with, 10 μM or 30 μM tBHQ for 24 h (a and c) or 30 μM tBHQ for different time periods as shown (b and d). Data represent mean ± SEM, analysed using one-way anova and Bonferroni *post hoc* test (*n* = 3, each concentration performed in triplicate) where *p < 0.05, **p < 0.01 ***p < 0.001 compared to vehicle control.

To ascertain whether the cytoplasmic retention of FoxO3a by tBHQ is accompanied by a decrease in FoxO activity, a luciferase assay was employed to examine changes in gene expression downstream of a forkhead-response element (FHRE-luc).To first define the experimental parameters, the effect of tBHQ on ARE activity were examined, as ARE is a well-characterized target of tBHQ (Kraft *et al.* 2004; Shih *et al.* 2005; Bahia *et al.* 2008). tBHQ caused a concentration- (Fig 3a) and time-dependent (Fig 3b) increase in ARE-mediated luciferase expression in neurones with maximal effects observed at 30 μM tBHQ for 24 h. In contrast, tBHQ caused a significant decrease in basal levels of FHRE activity with maximum effects again observed at 30 μM tBHQ for 24 h (Fig 3c and d).

### tBHQ inhibits NMDA-induced nuclear translocation of FoxO3a and reduces caspase 3A activation

**Fig 4:**
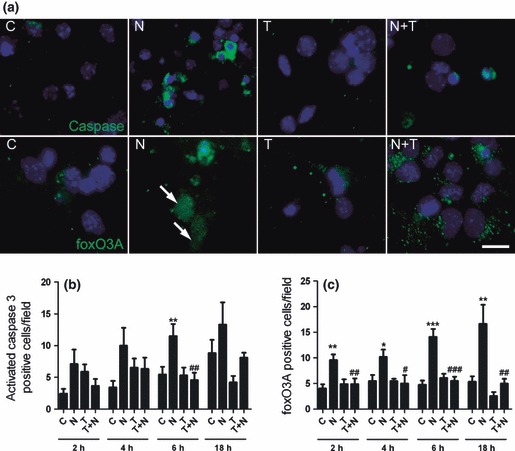
NMDA induction of activated caspase is accompanied by accumulation of nuclear FoxO3A and blocked by tert-Butylhydroquinone (tBHQ). (a) Representative photomicrograph showing cortical neurones (8DIV) after 6-h treatment with 0.1% ethanol vehicle (C), NMDA (50 μM) (N), tBHQ (30 μM) (T), or NMDA and tBHQ combined (N + T). Top panel shows staining for activated caspase 3 (green), and nuclear Hoe 33342 (blue). Bottom panel shows staining for foxO3A (green) and Hoe 33342 (blue), arrowheads indicate cells with strongly foxo3A-positive nuclei. Scale bar = 10 μm. (b) shows quantification of nuclei associated with activated caspase 3 at different time points after application of 0.1% ethanol vehicle (C), NMDA (50 μM) (N), tBHQ (30 μM) (T) or tBHQ and NMDA combined (T + N). (c) shows quantification of foxO3A-positive nuclei after the same treatments. In B + C, Bars show means and SEM derived from counts of six to nine fields from three experiments. Analysis was by anova and Bonferroni’s multiple comparison test: *^,^**^,^*** = *p* < 0.05, *p* < 0.01, *p* < 0.001 compared to vehicle control. ^#, ##, ###^ = *p* < 0.05, *p* < 0.01, *p* < 0.001 compared to NMDA treatment only.

We next tested if the induction of apoptotic markers following treatment with NMDA correlated with increase in nuclear FoxO3a and also whether NMDA-dependent caspase 3 activation and FoxO3a nuclear localization could be abrogated by tBHQ. As expected, NMDA (50 μM, 2–18 h) caused a time-dependent elevation in neurones containing activated caspase 3 (Fig 4a, top panel). Relative to vehicle controls, the number of cells containing activated caspase after NMDA treatment were 291 ± 93%, 290 ± 82%, 211 ± 35% and 151 ± 42% at 2, 4, 6 and 18 h, respectively (*n* = 6–9), which was statistically significant at the 6-h time point (Fig 4b). At all time points, the number of activated caspase-positive cells in the presence of tBHQ (30 μM) and NMDA (50 μM) was lower than the number of activated caspase-positive cells in the presence of NMDA alone, and at 6 h, the tBHQ reduction of NMDA-induced caspase activation was highly significant (40 ± 10% of NMDA-treated numbers, *n* = 9). The phenolic antioxidant tBHQ in its own right did not significantly decrease the number of caspase-positive cells at any time point. We note that, in these experiments, longer incubation times resulted in a time-dependent increase in the number of activated caspase 3-positive cells, even in the absence of any stimulation, suggesting some cell death in these cultures. For example, at 18 h, the vehicle control cultures had 361 ± 85% (*n* = 9) more caspase-positive cells compared with the vehicle control cultures at 2 h. In parallel with induction of the apoptotic marker, activated caspase 3, NMDA-induced nuclear foxO3A (Fig. 4a). The NMDA-induced increase in cells with nuclear foxO3A were 239 ± 27%, 188 ± 26%, 295 ± 32% and 312 ± 70% to the level in vehicle-treated controls, at 2, 4, 6 and 18 h respectively (Fig 4c, *n* = 9). This increase was statistically significant at all time points tested. At all time points, tBHQ prevented the NMDA-induced increase in foxo3A. Co-administration of tBHQ with NMDA significantly reduced in the number of cells with nuclear foxO3A to 51 ± 12%, 49 ± 16%, 39 ± 6% and 30 ± 6% to the levels with NMDA alone at 2, 4, 6 and 18 h respectively (Fig 4c, *n* = 6–9) tBHQ in its own right did not cause a significant change in the number of cells with nuclear foxO3A.

### tBHQ opposes NMDA-induced increase in forkhead-response element-activity and protects against NMDA-evoked toxicity

**Fig 5:**
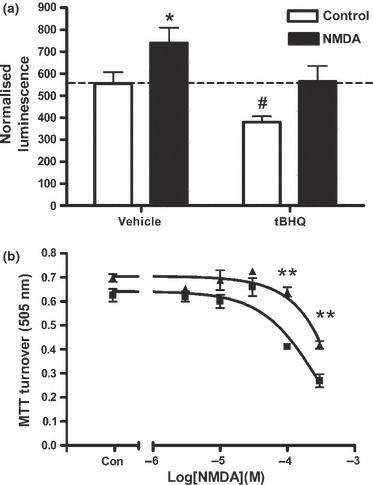
tert-Butylhydroquinone (tBHQ) opposes NMDA-induced up-regulation of FKHD-mediated gene expression and protects against NMDA-evoked neuronal damage. (a) Cortical neurones (8 DIV) were transfected with FHRE-luc and were then treated with NMDA (50 μM; 6 h;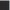) or vehicle control (□) in the presence and absence of tBHQ (30 μM). Data represent mean ± SEM, analysed using one-way anova and Bonferroni *post hoc* test (*n* = 3, each concentration performed in triplicate). NMDA increased FHRE activity compared with vehicle control *p < 0.05 and tBHQ inhibited FKHR compared to vehicle control ^#^*p* < 0.05. (b) Cultured cortical neurones (8 DIV) were incubated with increasing concentrations of NMDA in the presence of 30 μM tBHQ (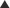) or vehicle control (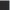) for 24 h. Cell viability was assessed using the MTT assay where the absorbance of reduced MTT was read at 505 nm. Neurones co-treated with tBHQ were significantly protected against 100 and 300 μM NMDA insults. Data represent mean ± SEM, analysed using one-way anova and the Bonferroni *post hoc* test (*n* = 4) where **p < 0.01 for individual NMDA concentrations versus control.

We further tested if tBHQ opposed NMDA effects via regulation of FHRE activity. Neurones were treated with NMDA (50 μM; 6 h) in the presence and absence of tBHQ (30 μM). NMDA increased FHRE activity consistent with the observed increase in nuclear FoxO3a and this increase was attenuated by co-treatment with tBHQ (30 μM) (Fig 5a). We note that longer term exposure (24 h) to NMDA (50 μM) induced neuronal loss, which confounded the luciferase expression data. Short-term exposure (20 min) to NMDA inhibited FHRE activity (data not shown), consistent with previous reports of pre-conditioning-dependent neuroprotection (Soriano *et al.* 2006).

Finally, to determine whether tBHQ actions can confer protection against excitotoxicity, neuronal viability was examined following exposure to NMDA (3–300 μM, 24 h) in the presence or absence of tBHQ (30 μM). Exposure to NMDA caused concentration-dependent neuronal cell damage as assessed by a reduction in MTT turnover. Co-treatment with tBHQ significantly inhibited NMDA-induced reduction in MTT turnover, consistent with tBHQ eliciting a neuroprotective effect. (Fig 5b).

## Discussion

FoxO3a activity is enhanced in neurones principally by a loss of trophic support (Gilley *et al.* 2003), but here we have shown that prolonged exposure of cortical neurones to NMDA also appears to enhance nuclear accumulation of FoxO3a. This is in general agreement with a recent report that extrasynaptic NMDA receptor stimulation enhances FoxO3a nuclear translocation in hippocampal neurones (Dick and Bading 2010). Enhanced nuclear translocation of FoxO3a is consistent with an NMDA-stimulated loss of PI3-kinase-dependent phosphorylation of FoxO3a, which has also been observed following exposure to high concentrations of glutamate (Zheng and Quirion 2009). Here, we show a correlation between NMDA-induced loss of FoxO3a phosphorylation and FoxO3 nuclear localization, which is reflected in an increase in FHRE-mediated gene expression, induction of a pro-apoptotic protein known to be regulated directly by FoxO proteins, FasL, and NMDA-induced cell death. In contrast, calcium-signalling evoked by stimulation of synaptic NMDA receptors has been shown to protect against FoxO3a translocation (Dick and Bading 2010), indeed, we observed inhibition of FHRE-mediated gene expression upon short-term exposure to NMDA (not shown), which is consistent with neuroprotective PI3-kinase-dependent Akt signalling activity associated with acute activation of this receptor population (Perkinton *et al.* 2002; Soriano *et al.* 2006). It is likely, therefore, that the switch to increased FHRE-mediated gene expression and pro-death signalling observed following prolonged exposure to NMDA results from down-regulation of the protective PI3-kinase-dependent element of the pathway and recruitment of the extrasynaptic NMDA receptor population in a similar manner to the well-characterized CREB shut-off pathway (Hardingham and Bading 2010).

As tBHQ as well as various dietary polyphenolics stimulate PI3-kinase-dependent Akt phosphorylation and up-regulate Nrf2-mediated antioxidant response element activity (Kang *et al.* 2002; Kraft *et al.* 2004; Schroeter *et al.* 2007; Bahia *et al.* 2008; Arredondo *et al.* 2010), and tBHQ has been reported to protect motor neurones against FasL-induced neurodegeneration (Pehar *et al.* 2007), we considered that tBHQ might also suppress FoxO3a nuclear translocation, which might underlie some of its neuroprotective efficacy. Accordingly, we found that tBHQ stimulated PI3-kinase-dependent phosphorylation of FoxO3a in neurones and this correlated with its retention in the cytosol against a basal low-level accumulation of FoxO3a in the nucleus. Furthermore, tBHQ effectively suppressed NMDA-dependent FoxO3a nuclear accumulation and FHRE activity, attenuated NMDA-dependent activation of caspase 3 in neurones and reduced NMDA-induced neuronal death. Although these observations are strictly correlative, they are strongly suggestive of a very close relationship between regulation of FoxO3a activity and neuronal cell death and identify tBHQ and compounds of this type, for example dietary polyphenolic compounds, as potential suppressors of FoxO-dependent cell death. Our findings have been carried out in neuronal cultures with very few astrocytes present (< 5%), suggesting that antioxidant signalling to prevent FoxO activation and neuronal death are mediated by pathways intrinsic to neurones, nevertheless we cannot completely exclude at this time that tBHQ is acting on astrocytes to confer neuronal protection. The precise mechanisms underlying these protective effects are not yet clear, but there is emerging evidence that deacetylation and methylation regulate the stability and activity of FoxO3 (Wang *et al.* 2012). Most notably, oxidative stress-evoked neuronal apoptosis is inhibited by Set9-mediated lysine methylation of FoxO3 (Xie *et al.* 2012) and this therefore represents a potential target for the protective actions of tBHQ and dietary antioxidants.

Our study supports emerging data, which suggest that FoxO3A is a key mediator of the progressive neuronal cell loss associated with neurodegeneration and with other disorders of the nervous system. For example, increased FoxO3a has been found localized to Lewy bodies and Lewy neurites in Parkinson’s disease brain tissue (Su *et al.* 2009) and activation of FoxO by LRRK2, associated with autosomal-dominant late-onset Parkinson’s disease, enhances neuronal cell death in *Drosophila* (Kanao *et al.* 2010). With respect to Alzheimer’s disease, inactivation of FoxO3a activity is correlated with reduced Alzheimer’s disease-like pathology and with preservation of spatial reference memory in Tg2576 mice (Qin *et al.* 2008). This implies that APP over-expression activates FoxO3a in brain regions associated with spatial memory, which is consistent with the reduced levels of Akt phosphorylation observed in the hippocampus of APPswe mutant mice (Abbott *et al.* 2008). In motor neurons, FoxO3a activity is thought to contribute to neuronal cell death (Brunet *et al.* 1999; Barthelemy *et al.* 2004) and similarly we interpret our observed increase in nuclear FoxO3a in motor neurons as a contributing factor to apoptosis; however, this is not consistent with findings that stimulating FoxO3a activity is broadly neuroprotective in *C-elegans*, *Drosophila* and in mouse motor neurons (Mojsilovic-Petrovic *et al.* 2009). It is therefore possible that the enhanced nuclear levels of FoxO3a observed in our motor neurons represent a failed neuroprotective response. Generally, differences in beneficial versus harmful effects of FoxO3a are more likely to relate to cell-circumstance-specific level of expression, activation and post-translational modification (Mojsilovic-Petrovic *et al.* 2009).

On the basis of our data, we propose, therefore, that tBHQ and perhaps other dietary polyphenolics, in addition to their well-documented effects on antioxidant response element-mediated gene expression, exert neuroprotection by regulating the localization and activity of FoxO3a, thereby opposing apoptosis. In this context, we note that a number of flavonoids are also capable of modestly reducing FHRE activity (data not shown), suggesting that regulation of nuclear translocation of FoxO3A might be a common feature of molecules possessing antioxidant activity. Indeed, our data predict that activation of the antioxidant response element is accompanied by reciprocal regulation of FHRE. We do not yet know if these observations are mechanistically related, but hypoxic pre-conditioning which up-regulates an antioxidant response protects against transient global ischaemia by increasing FoxO phosphorylation (Zhan *et al.* 2010) and signalling pathways linking oxidative stress to neuronal cell death have previously been attributed to FoxO activation (Lehtinen *et al.* 2006; Yuan *et al.* 2009), so the link between these responses is compelling. In summary, our data support a role for FoxO3a in NMDA-evoked neuronal apoptosis and suggest that phenolic antioxidants such as tBHQ oppose stress-induced activation of FoxO3a and therefore may have potential neuroprotective utility in neurodegeneration.

## Abbreviations

BSA: bovine serum albumin
FHRE: Forkhead-response element
FoxO: Forkhead transcription factor
PBS: phosphate-buffered saline
tBHQ: tert-Butylhydroquinone

## Supporting information

Figure S1 Kainate drives nuclear accumulation of Foxo3a in motor neurones. As a service to our authors and readers, this journal provides supporting information supplied by the authors. Such materials are peer-reviewed and may be re-organized for online delivery, but are not copy-edited or typeset. Technical support issues arising from supporting information (other than missing files) should be addressed to the authors.
