# Supplementary material for: Neuroprotective effects of phenolic antioxidant tBHQ associate with inhibition of FoxO3a nuclear translocation and activity
Source: J Neurochem. 2012 Oct;123(1):182–91. doi: 10.1111/j.1471-4159.2012.07877.x (PMC3494983; doi:10.1111/j.1471-4159.2012.07877.x)
Supplement: Figure S1 — Kainate drives nuclear accumulation of Foxo3a in motor neurones. As a service to our authors and readers, this journal provides supporting information supplied by the authors. Such materials are peer-reviewed and may be re-organized for online delivery, but are not copy-edited or typeset. Technical support issues arising from supporting information (other than missing files) should be addressed to the authors. [file jnc0123-0182-SD1.pdf]

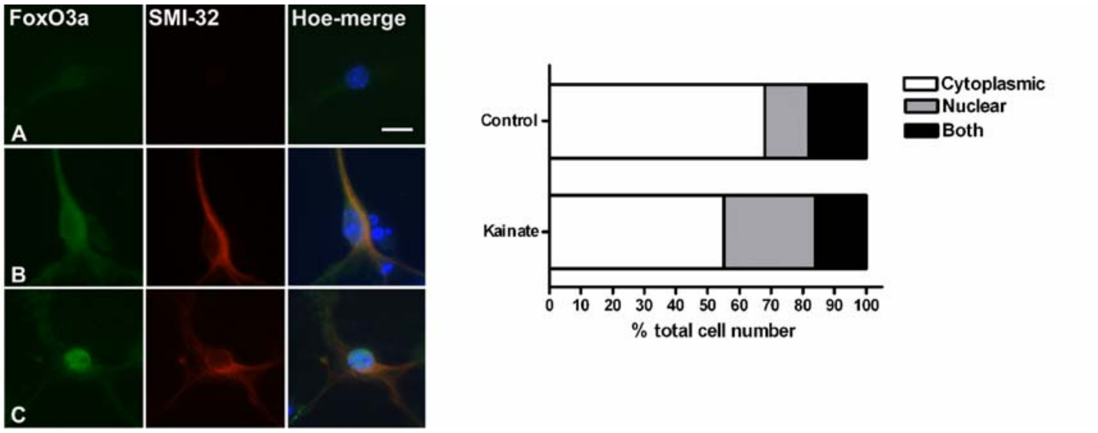

Supplementary 1. Kainate drives nuclear accumulation of Foxo3a in motor neurones.

Motor neurone cultures treated with 50 μM kainate for 2 h were fixed and stained with anti-SMI-32 to label motor neurones (red), Foxo3a (green) and Hoescht (blue). Negative controls (**A**) were stained with secondary antibody alone. Representative images of the cellular localisation of Foxo3a following vehicle (B) or kainate treatment (C) are depicted. Scalebar = 20 μm. The total number of cells in each category was counted following treatment with vehicle or kainate. Not all motor neurones showed nuclear FoxO3a localisation post-agonist treatment so, to assess the extent of translocation, cells were separated into three groups according to whether immunoreactivity was predominantly cytoplasmic (empty bar), predominantly nuclear (grey bar) or distributed between both compartments (black bar).

Approximately 30% of control cells showed at least some FoxO3a immunoreactivity in the nucleus. In motor neurones treated with kainate the proportion of cells showing nuclear FoxO3a staining increased to 41 %. The proportion of cells exhibiting predominantly nuclear FoxO3a staining increased from 14% to 29%.
